# Tuning the performance of vanadium redox flow batteries by modifying the structural defects of the carbon felt electrode

**DOI:** 10.3762/bjnano.10.165

**Published:** 2019-08-13

**Authors:** Ditty Dixon, Deepu Joseph Babu, Aiswarya Bhaskar, Hans-Michael Bruns, Joerg J Schneider, Frieder Scheiba, Helmut Ehrenberg

**Affiliations:** 1 Central Electrochemical Research Institute (CSIR-CECRI), Karaikudi, 630003, India; 2 Fachbereich Chemie, Eduard-Zintl Institut für Anorganische und Physikalische Chemie, Technische Universität Darmstadt, Alarich-Weiss-Straße 12, 64287 Darmstadt, Germany; 3 Institute for Applied Materials - Energy Storage Systems (IAM-ESS), Karlsruhe Institute of Technology (KIT), Hermann-von-Helmholtz-Platz 1, 76344 Eggenstein-Leopoldshafen, Germany

**Keywords:** carbon felt, defects, nitrogen plasma, vanadium redox flow battery (VRFB)

## Abstract

Polyacrylonitrile (PAN)-based carbon felt was subjected to N_2_-plasma treatment to increase the heteroatom defects and reactive edge sites as a method to increase the performance in vanadium redox flow batteries (VRFBs). N-doping in the felt was mainly in the form of pyrrolic and pyridinic nitrogen. Even though the amount of oxygen functional groups on the N_2_-plasma-treated sample was very low, the felt showed enhanced electrochemical performance for both V^3+^/V^2+^ as well as V^5+^/V^4+^ redox reactions. The result is highly significant as the pristine electrode with the same amount of oxygen functional groups showed significantly less activity for the V^3+^/V^2+^ redox reaction. Overall, the single-flow cell experiments with N_2_-plasma-treated felt showed superior performance compared to the pristine sample. Therefore, the enhanced performance observed for the N_2_-plasma-treated sample should be attributed to the increase in defects and edge sites. Thus, from the present study, it can be concluded that an alternate way to increase the performance of the VRFBs is to introduce specific defects such as N-doping/substitution or to increase the edge sites. In other words, defects induced in the carbon felt such as heteroatom doping are as beneficial as the presence of oxygen functional groups for the improved performance of VRFBs. Therefore, for an optimum performance of VRFBs, defects such as N-substitution as well as oxygen functionality should be tuned.

## Introduction

In every part of the world, the contribution of electrical energy harvested from a renewable source, such as wind, photovoltaics, etc., to the electrical grid system is increasing. In contrast to electric energy production from fossil or nuclear fuels, the generation of energy from renewable sources is intermittent by nature. The intermittent nature of such energy production can lead to the destabilization of the grid. This issue demands the development of durable and efficient electrical energy storage systems which can store the excess electrical energy from renewable energy sources during peak production and supply the stored energy to the grid during a depletion in the production. In this context, the all-vanadium redox flow battery (VRFB) is one of the most promising and flexible stationary electrical energy storage systems. Unlike Pb acid, Li-ion batteries or even flow batteries like zinc/bromine, the electrical energy in VRFBs is completely stored by the electrolyte in an external tank. Thus, in VRFB systems, the power and energy can be decoupled, that is, to store more energy, only the tank size needs to be increased. Moreover, since the system uses only a single redox species, element cross-contamination issues, which are common in other redox flow batteries such as Cr/Fe, are obviously nonexistent [1]. Nevertheless, the system suffers from irreversible capacity fade due to parasitic reactions such as air oxidation of V^2+^ species and hydrogen evolution reaction (HER) at the negative electrode [2–4]. The air oxidation of V^2+^ species can be completely prevented by keeping the negative tank under inert gas atmosphere. However, the HER at the negative electrode is almost unavoidable as the redox potential of V^3+^/V^2+^ (−0.26 V vs normal hydrogen electrode (NHE)) reaction is very close to HER (0 V vs NHE). To minimize the HER, the negative electrode surface structure should be tuned in such a way that it tends to preferably bind V^3+^/V^2+^ ions over H^+^ ions. Creating oxygen functional groups on the surface of the anode is one way to achieve this [5–6]. Langner et al. have shown that on a functionalized electrode, in the presence of V^3+^, the HER is suppressed as the V^3+^ ions get preferentially bonded to the oxygen functional groups [6]. Furthermore, they proposed that it is essential to have at least 5% oxygen functionality on the surface of the carbon felt for the unhindered reduction of V^3+^ ions. In fact, carbon felt with a surface coverage as high as 23% oxygen functionality showed relatively enhanced VRFB performance [7]. Nevertheless, the above-mentioned electrode with a higher amount of oxygen functional groups, when used in a three-electrode configuration, showed poor electrochemical performance for the positive (V^5+^/V^4+^) redox reaction. Taking into account that the negative redox reaction is the limiting reaction in VRFB, the overall enhancement in the full cell performance was purely attributed to enhancement in the V^3+^/V^2+^ redox kinetics due to the presence of functional groups [8]. Thus, it is extremely important to optimize and maintain the amount of functional groups on the surface of the carbon felt especially when used as a negative electrode in VRFB. However, it has been found that due to electrochemical as well as chemical ageing, both electrode surfaces tend to oxidize with the additional formation of oxygen functional groups [9–10]. Excess oxidation of the carbon felt can also introduce nonselective functional groups such as –C–O and –C=O and reduces the sp^2^ carbon content or the graphite content of the felt. The formation of nonselective functional groups can impede the redox reaction. For example, Estevez et al. showed that the presence of –O–C=O groups increases the performance of the VRFB whereas the presence of –C–O and –C=O degrades it [11]. In the long run, reduction in the graphite or sp^2^ carbon content of the felt reduces the electrical conductivity, leading to performance loss. Furthermore, it has been proposed by Schweiss et al. that an increase in the amorphous content in the felt can increase the hydrogen evolution reaction [12]. In one way or the other, functionalization with heteroatoms will always reduce the graphitic nature as functionalization proceeds by breaking of the C_6_ rings, and in many cases, with the formation of a sp^3^ hybridized carbon atoms (out of plane with the graphene layer). Moreover, most of these functional groups will be predominantly formed at graphite edge sites which are much more active than a basal carbon [13]. Therefore, to obtain reasonable VRFB performance, the carbon edge sites of the carbon felt electrode should be preserved or the functional group formed on this site by chemical or electrochemical ageing should promote the redox reaction. Another possible way to create a reaction site or catalytic center in graphite is by doping it with heteroatoms such as B, N, or P. The heteroatom perturbs the electronic structure of the graphite layer subjected to doping, leading to enhanced polarization [14]. N-doped carbon-based electrodes have been successfully tested in VRFBs. For example, Wang et al. developed carbon felt deposited with N-doped carbon nanotubes which showed enhanced VRFB performance [15]. He et al. produced N-doped carbon felt by heating the commercial felt at 600 and 900 °C in the presence of NH_3_ gas. This felt showed enhanced VRFB performance, owing to the increase in electrical conductivity as well as active sites [16]. In this work, a carbon felt electrode with minimum oxygen functional groups and a larger amount of defects in the form of N-doping and edge sites was prepared by employing the N_2_ plasma technique. The N_2_-plasma-treated sample showed enhanced electrochemical performance in a VRFB compared to the untreated sample with fewer defects. The commercial carbon felts (GFD-type) used as electrode materials in the present study are made out of a polyacrylonitrile (PAN) precursor. In contrast to the commonly employed thermal activation process, the plasma treatment process is quick, and subsequent physical or chemical changes incurred will be uniform across the felt. Apart from that, it is observed that the surface area of the material is not affected by the plasma treatment process.

## Results and Discussion

**Figure 1:**
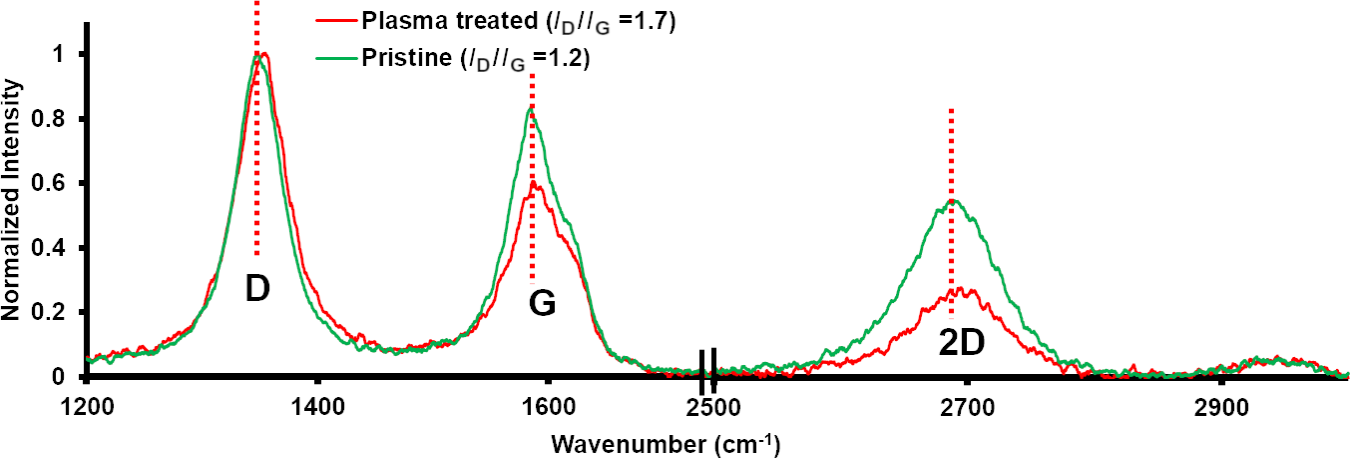
Raman spectra obtained for pristine as well as N_2_-plasma-treated sample.

In this work, the N_2_ plasma treatment process is applied to PAN-based felts to increase the amount of defects. The normalized spectra obtained for the Raman measurements are shown in Figure 1. In order to investigate the degree of graphitization and defects formed during the plasma treatment process, the intensity of the G- and D-band centered at 1590 cm^−1^ and 1356 cm^−1^ are compared. The G-band in graphitic material arises from the in-plane vibration of sp^2^ carbon atoms. Whereas the D-band arises from out-of-plane vibrations from carbon associated with defects. Therefore, the ratio of the intensity of the D- and G-bands (*I*_D_/*I*_G_) gives direct information about the extent of defects in a graphite material [17–18]. From the Raman spectral analysis, it was found that the pristine sample had a lower *I*_D_/*I*_G_ ratio of 1.2 compared to the N_2_-plasma-treated sample of 1.7. This indicates that the N_2_ plasma treatment process served to increase the defects in the carbon felt. Moreover, the D-band of the plasma-treated sample was shifted to a higher frequency, indicating an increase in the defect density. Mostly, this increase in defects can be correlated to heteroatom substitution/doping (N-doping) and the simultaneous creation of new edge sites [17]. At 2690 cm^−1^ a symmetric second order D-band (2D) is visible for both samples. Careful analysis reveals that the 2D peak intensity is lower for the plasma-treated sample, indicating possible doping [17].

**Figure 2:**
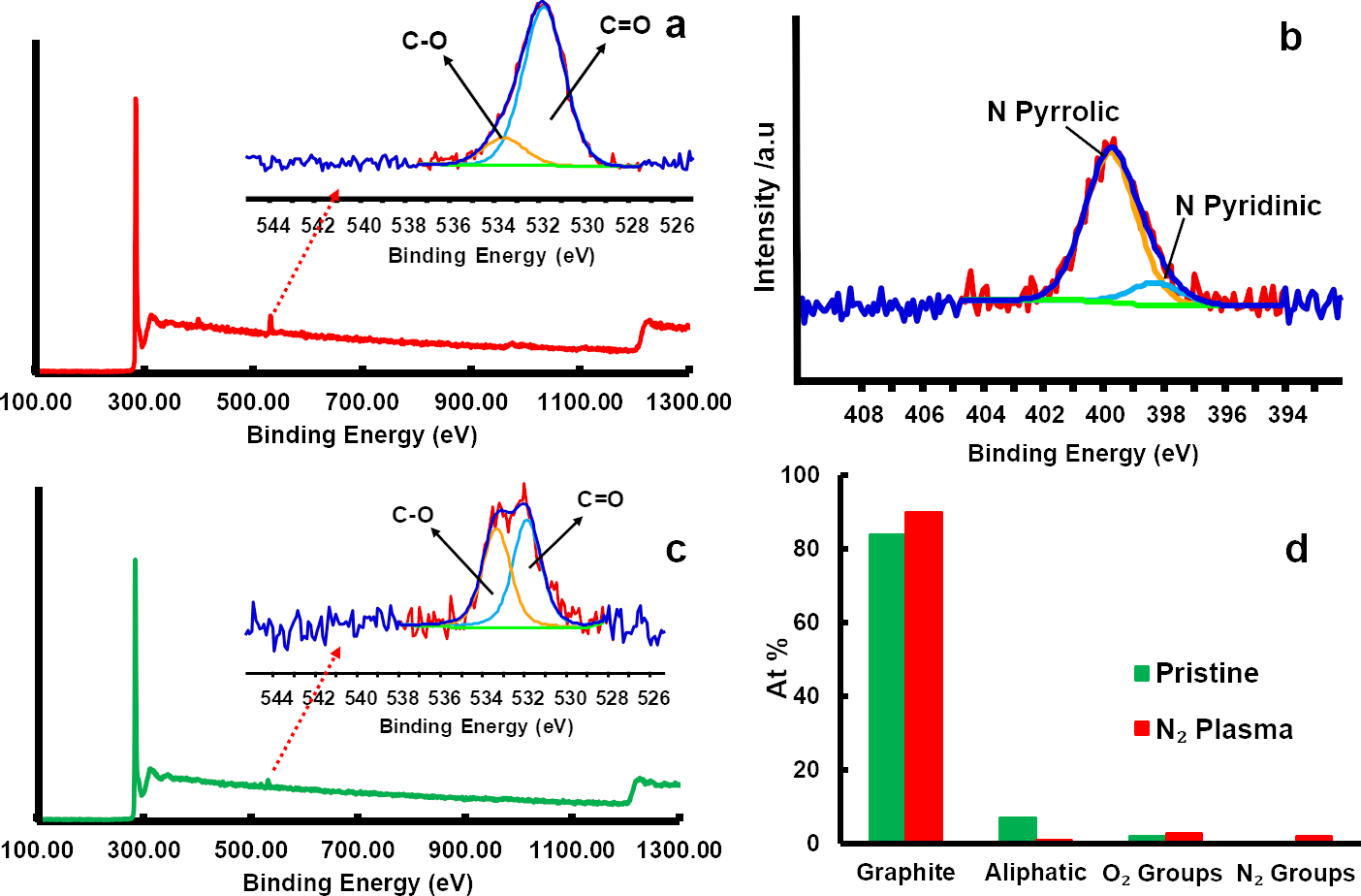
XPS results obtained for pristine and N_2_-plasma-treated samples. a) Survey scan for the N_2_-plasma-treated sample and inset showing the O 1s peak fitting results, b) N 1s peak fitting for the N_2_-plasma-treated sample, c) survey scan for the pristine sample and inset showing the O 1s peak fitting results, and d) composition of the various groups obtained after peak fitting.

In order to investigate the N-doping in a plasma-treated felt, X-ray photoelectron spectroscopy (XPS) analysis of the samples was carried out. The N_2_-plasma-treated sample was characterized by a N 1s peak with maxima around 399 eV. The N 1s peak could be deconvoluted into pyridinic and pyrrolic N contributions, with maxima at 398.3 and 399.8 eV, respectively. The XPS results, as well as the quantification of various groups on the surface of the felt are shown in Figure 2. Thus, from these results, it can be concluded that N_2_ plasma treatment can be applied to induce N-doping defects. Both pyrrolic as well as pyridinic N observed in the XPS spectra are incorporated into the graphene layer [19–20]. The doping of N as observed here differs from the functionalization by the fact that during the doping process, the N atom is directly bonded with other carbon atoms inside the graphene framework, whereas during the functionalization process, the nitrogen groups are bonded on one of the edge sites of the carbon atoms. Since XPS shows only the presence of pyrrolic and pyridinic contributions, any N-functionalization can be ruled out [20–21]. In contrast to oxygen, nitrogen is less reactive, and its atomic size is close to carbon. Therefore, with N_2_ plasma treatment, doping prevails over functionalization. The elementary composition from the XPS analysis revealed the presence of approximately 2% of nitrogen on the surface of the felt, which translates into a substantial amount of N doping, given that a N-doping level as low as 1 atom % can have a significant effect on the electronic structure of carbon materials [22].

**Figure 3:**
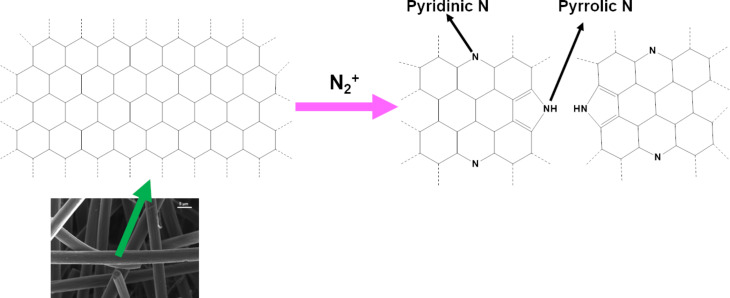
Schematic representation of N-doping induced by N_2_ plasma treatment in a graphite lattice.

Additionally, from the XPS analysis, approximately 2% and 3% of oxygen functional groups (C=O and C–O) was found to be present on both pristine as well as plasma-treated samples, respectively. Furthermore, it can also be seen from the XPS results that, compared to the pristine sample, the N_2_-plasma-treated sample has the highest graphitic content on the surface. This result contradicts the Raman spectroscopy result where the plasma-treated sample in fact showed more defects. It must be emphasized that in the present work no peak fitting was carried out on the C 1s peak to quantify the defects. It may be stressed that the graphite content obtained from XPS also has a contribution from the defects. The increase in the graphitic amount could be correlated to the corresponding decrease in the amount of aliphatic carbon. The source of this aliphatic carbon is either from the graphitization process of the PAN fibers or simply the atmospheric ageing of the felt. It is already known from the literature that graphitization or atmospheric ageing can leave some aliphatic or polyaromatic tar-like residues on the surface of the felt [6,23]. Thus, it can be concluded that apart from inducing N-doping, the N_2_ plasma treatment also increased the apparent graphite amount on the surface by removing the aliphatic groups (C–H and C–C) from the surface of the fibers. The schematic representation of N-doping induced by the N_2_ plasma treatment in a graphite lattice is shown in Figure 3.

**Figure 4:**
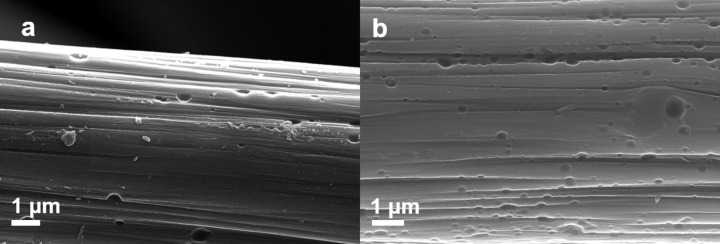
SEM images of a) pristine and b) N_2_-plasma-treated samples.

From the scanning electron microscopy (SEM) analysis, it can be seen that the surface morphology of the fibers of both samples looked identical and thus any kind of surface roughening leading to an increase of the surface area can be ignored. This is further supported by our previous study were the BET measurements did not show any increase in surface area for oxygen-plasma-treated samples [7]. The SEM images of the pristine as well as the N_2_-plasma-treated samples are shown in Figure 4.

**Figure 5:**
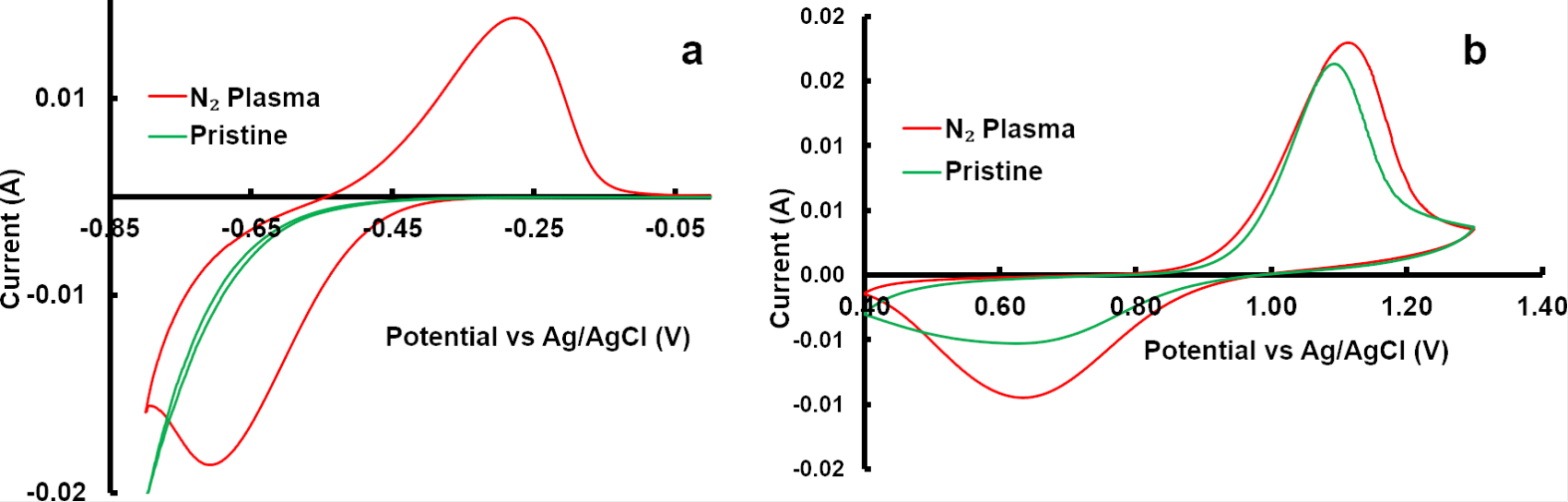
CV curves obtained for the pristine and the N_2_-plasma-treated sample: a) negative redox reaction, b) positive redox reaction.

In order to evaluate the electrochemical performance of the N_2_-plasma-treated sample, cyclic voltammetry (CV) measurements were carried out. In contrast to the pristine sample, a prominent V^3+^/V^2+^ redox peak is observed for the N_2_-plasma-treated sample. The CV of the pristine sample is mainly characterized by a hydrogen evolution peak. The CV curves for both negative and positive redox reactions are shown in Figure 5.

**Figure 6:**
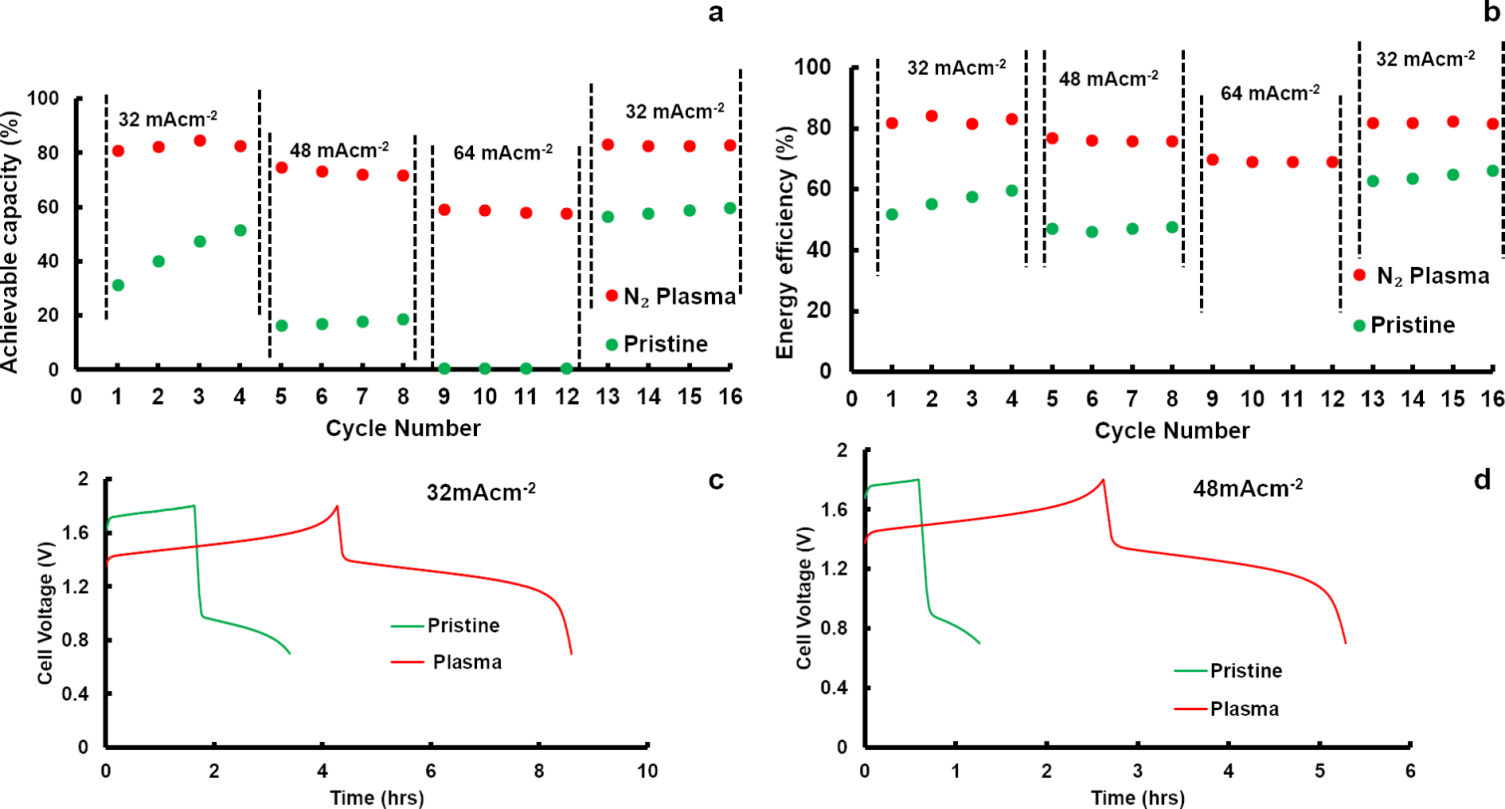
Single-cell measurement results with pristine (green) and N_2_-plasma-treated samples (red). a) Maximum discharge capacity obtained during cycling, b) energy efficiency achieved during cycling, c) charge–discharge curve obtained for cycling at 32 mA cm^−2^, d) charge–discharge curve obtained for cycling at 48 mA cm^−2^.

Given that the pristine and N_2_-plasma-treated samples have almost the same amount of oxygen functional groups, the enhanced activity shown by N_2_-plasma-treated samples towards the V^3+^/V^2+^ redox reaction should be attributed to the specific defects such as N-doping and the increase of the edge sites. More recently, Xu et al. showed, using first-principle calculations, that N-doping (especially the pyridinic and pyrrolic forms) enhances the water adsorption or hydrophilicity of the graphite electrode [24]. Thus, it may be concluded that the pyridinic and pyrrolic nitrogen formed during the N_2_-plasma process enhanced the wettability of the felt, which in turn facilitates the adsorption of the V^2+^/V^3+^ ions. Apart from defects in the form of N-doping, a higher amount of edge sites formed during the N_2_-plasma process also influences the V^3+^/V^2+^ redox reaction. This is because the half-cell reaction is known to depend greatly on the carbon edge sites [25]. Another reason for the enhanced activity of the N_2_-plasma-treated samples towards the V^3+^/V^2+^ reaction is that the aliphatic carbonaceous materials on the surface of the felts are removed during the N_2_-plasma process. As a result of this process, more electrochemically active sites (edge sites) are available for the V^3+^/V^2+^ redox reaction. As far as the V^5+^/V^4+^ redox reaction (i.e., the positive redox reaction) is concerned, both pristine, as well as N_2_-plasma-treated carbon felts showed electrochemical activity. Compared to pristine carbon felt the N_2_-plasma-treated sample showed an earlier onset potential for the V^5+^/V^4+^ redox reaction. Thermodynamically, the V^4+^ to V^5+^ redox reaction takes place at 1.0 V vs a normal hydrogen electrode (NHE). Taking into account that carbon oxidation is feasible at potentials as low as 207 mV vs NHE [26], the higher potential of positive electrode of the VRFB can accelerate the carbon oxidation. During the carbon oxidation process, oxygen functional groups can be formed as an intermediate on the carbon electrode [27–28]. Therefore, oxygen functional groups can be formed on the surface of the felt, and especially on the positive electrode as it experiences a relatively higher potential. In fact, Derr et al. have observed an increase in the amount of functional groups on the surface of both negative and positive carbon felt electrodes after prolonged cycling [9]. Given that a higher potential is favorable for carbon oxidation, it can be concluded that the presence of functional groups on the carbon felt is not a prerequisite for the onset of the V^5+^/V^4+^ redox reaction. CV studies were further supported by full cell studies, where the electrochemical performance of the N_2_-plasma-treated sample was superior to the pristine sample. The cell with the N_2_-plasma-treated sample showed a higher energy efficiency and delivered higher capacities at all investigated current densities. The single-cell measurement results are shown in Figure 6. With the pristine sample at higher current densities (≥64 mA cm^−2^), the cell ran into HER. Since the negative electrode reaction is the performance-limiting reaction, the superior performance observed for the single-cell measurement could be attributed to the enhancement in the V^2+^/V^3+^ redox reaction. However, it could also be seen that the performance of the pristine and the N_2_-plasma-treated sample improves with further cycling, and during the final cycle (13–16) the cells delivered higher capacities compared to the initial cycles (1–4).

**Figure 7:**
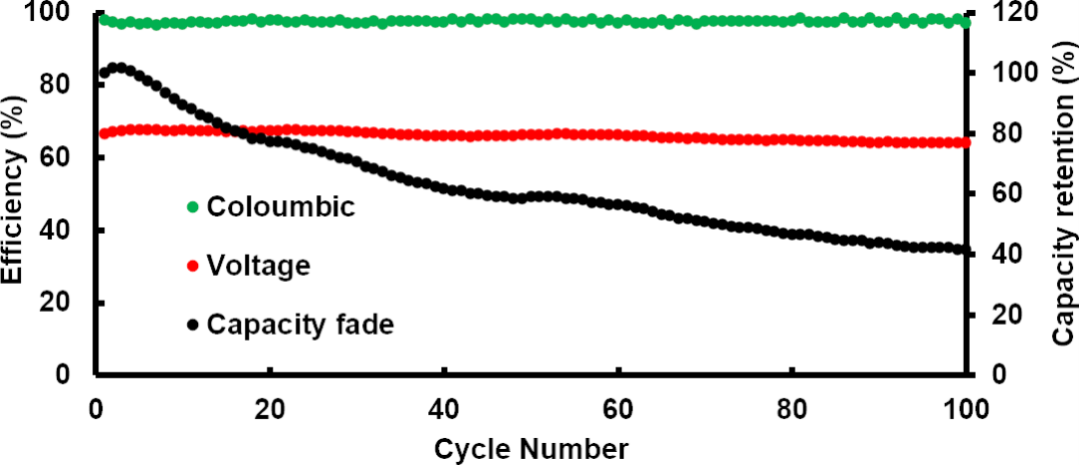
Evolution of efficiency and capacity retention during long-term cycling with a N_2_-plasma-treated sample.

This increase in performance can be attributed to oxygen functional groups (both hydroxyl and carboxyl) formed on the surface of the felt due to the chemical and the electrochemical ageing process as elucidated by Derr et al. [9]. Nevertheless, a capacity fade was observed on the N_2_-plasma-treated sample after long-term cycling at a current density of 80 mA cm^−2^ (see Figure 7). Capacity fade can be partially attributed to the HER taking place at the negative electrode due to the very low amount of oxygen functional groups. Moreover, the Nafion 117 membrane used for higher current density cycling leads to an electrolyte imbalance. A detailed investigation is still required to understand the overall mechanism of the capacity fade.

In a previous study [7], a similar extent of capacity fade was observed for heat-treated GFD (graphite-based) carbon felt samples with a higher amount of oxygen functional groups and a larger surface area than in this study. Nevertheless, the energy efficiency of the cell with the N_2_-plasma-treated electrode is higher than that with the heat-treated electrode. Therefore, for achieving the optimum VRFB performance, the electrodes, especially the anode, must be tuned for defects such as N-substitution as well as oxygen functionality (specifically –O–C=O groups). The present study predicts that the combination of various plasma techniques (O_2_/N_2_) and thermal activation could produce an ideal electrode for the anode in VRFB.

## Conclusion

When PAN-based GFD-type felts are subjected to N_2_ plasma treatment, defects are formed on the carbon felt. In addition to the increase in the amount of reactive edge sites, also heteroatom defects involving N-doping are created by the N_2_ plasma treatment. The surface of the plasma-treated samples was characterized by the presence of pyrrolic and pyridinic nitrogen. The N_2_-plasma-treated felt showed enhanced electrochemical performance compared to the pristine felt. Since both the pristine as well as the N_2_-plasma-treated sample had almost the same amount of oxygen functional groups, the superior performance observed for the former one is attributed to the additional defects formed during the plasma treatment. Nevertheless, the cell operated with the N_2_-plasma-treated sample suffered from capacity fade, which can most likely be attributed to hydrogen evolution at the negative electrode. Therefore, it may be concluded that for the optimum performance of the VRFB, a balance should be found between the amount of various defects such as heteroatom doping, edge sites and functional groups.

## Experimental

### Plasma treatment process

The plasma treatment was carried out on a pristine GFD-type carbon felt, obtained from SGL Carbon (SIGRACELL GFD3 EA), having a thickness of 3 mm, in a radiofrequency (rf) 13.56 MHz plasma setup (Femto, Diener electronic GmbH, Germany) [28]. The power rating of the rf generator is 300 W (max. rf power limited to 200 W). 5 × 5 cm felts were loaded into the plasma chamber which was subsequently evacuated to a pressure below 0.2 mbar before the chamber was filled with about 0.8 mbar of nitrogen. All plasma treatments were carried out for 40 min at 20% of the maximum power. In the present work a capacitively coupled parallel plate rf plasma was used. The separation between the plate was 10 cm and the samples were always placed on the bottom plate without any further connections.

### Raman spectroscopy

Raman measurements were carried out using a HORIBA (model: LabRAM HR Evolution) Raman spectrometer and microscope. An Oxxius 532 nm laser (100 mW) and a 50× optical lens were used to obtain the spectra. The spectra were recorded between 500 cm^−1^ to 3000 cm^−1^. The spectra were recorded with an acquisition time of 3 s. To prevent sample damage, the laser power was reduced to 10%.

### X-ray photoelectron spectroscopy (XPS)

XPS measurements were performed using a K-Alpha XPS instrument (Thermo Fisher Scientific, East Grinstead, UK). The data acquisition and processing using the Thermo Advantage software is described elsewhere [29]. All samples were analyzed using a focused (30–400 µm spot size), monochromatic Al Kα X-ray source. The Kα charge compensation system was employed during the experiment, using electrons of 8 eV energy and low-energy argon ions to prevent any localized charge build-up. The spectra were fitted with one or more Voigt profiles (binding energy (BE) uncertainty: ±0.2 eV). The analyzer transmission function, Scofield sensitivity factors [29], and effective attenuation lengths (EALs) for photoelectrons were applied for quantification. The EALs were calculated using the standard TPP-2M formalism [30]. All spectra were referenced to the C 1s peak of hydrocarbon at 285.0 eV binding energy, controlled by means of the well-known photoelectron peaks of metallic Cu, Ag, and Au.

### Scanning electron microscopy (SEM)

The carbon surface fiber morphology was investigated in a Zeiss Supra 55 SEM with primary electron energies of 5 keV and 15 keV and an in-lens detector.

### Electrochemical measurements

In this work, commercial carbon felts obtained from SGL Carbon in pristine form and after N_2_ plasma treatment were used as electrode materials. Cyclic voltammetry (CV) was performed in a three-electrode setup using a Reference 3000 instrument from Gamry with a Ag/AgCl reference electrode and a platinum mesh as a counter electrode. A modified configuration developed by Fink et al. was used as the setup for the working electrode (WE) [31]. The configuration was modified so that punched-out disks (Ø = 6 mm) of the felts attached to a glassy carbon rod were used as the WE. To achieve a better electrical contact, the punched-out felt was pierced through the middle by 5 cm long glassy carbon rod with a diameter of 1 mm. The positive half-cell reaction was measured in 0.1 molar vanadyl sulphate (Alfa Aeser) dissolved in 2 molar sulfuric acid (Sigma Aldrich). In order to obtain the V^3+^ electrolyte for the negative half-cell reaction, both tanks were filled with the same volume of the V^4+^ electrolyte and then potentiostatically charged at 1.7 V in a 10 cm^−2^ flow cell. The reduction to V^3+^ was determined as complete when the charging current reached less than 10 mA cm^−2^. All the cyclic voltammetry measurements were carried out at a scan rate of 5 mV s^−1^.

Single-cell measurements were performed using a modified direct methanol fuel cell from ElectroChem, having a pin-type flow field with an active area of 25 cm^2^. Additionally, a 2 mm Viton gasket was used as a spacer around the flow fields to achieve uniform compression. A commercial electrolyte from GFE GmbH Germany with 1.6 M vanadium salt (50/50 mol/mol V^4+^/V^3+^) and an anion exchange membrane, VX 20 from Fumatech was used for most of the single-cell experiments. Galvanostatic cycling was carried out using a single-cell test bench from Scribner (875 Redox Flow Cell Test System) at current densities of 32, 48, and 64 mA cm^−2^. Both electrolyte tanks were filled with 100 mL of electrolyte and the negative tank was always kept under nitrogen gas flow. Cut-off voltages of 1.8 V and 0.7 V were used for the charging and discharging steps, respectively. Between the charging and discharging, the cell was kept at an open-circuit voltage condition for 5 min. Long-term cycling measurements were carried out at a current density of 80 mA cm^−2^ with fresh electrolyte and fresh electrode and activated Nafion 117 membrane. In all the experiments, the electrolyte flow rate was kept at 100 mL min^−1^. The temperature of the cell and electrolyte was maintained at 22 °C throughout the electrochemical investigations.
